# Immune Checkpoint Inhibitors in Peripheral T-Cell Lymphoma

**DOI:** 10.3389/fphar.2022.869488

**Published:** 2022-04-26

**Authors:** Xi Chen, Wanchun Wu, Wenwen Wei, Liqun Zou

**Affiliations:** ^1^ Department of Radiotherapy, Cancer Center, West China Hospital of Sichuan University, Chengdu, China; ^2^ Department of Medical Oncology, Cancer Center, West China Hospital of Sichuan University, Chengdu, China

**Keywords:** peripheral T-cell lymphoma, immune checkpoint inhibitor, programmed cell death protein 1, expression, immunotherapy

## Abstract

Peripheral T-cell lymphomas (PTCLs) are highly heterogeneous and present significant treatment challenges. Immune checkpoint therapies, such as PD-1 and CTLA-4 inhibitors, have significantly changed the clinical management paradigm of tumors. The roles of immune checkpoints in PTCL and related agents have been actively explored over recent years. PD-1 and PD-L1 expression is detectable in both PTCL and immune cells within the tumor microenvironment and forms the basis for the exploration of antibodies targeting these proteins. Such antibodies are currently being investigated in clinical trials to guide individualized therapy. PD-1/PD-L1 inhibitors alone and in combination with chemotherapy, radiotherapy, or targeted therapy have shown broad clinical efficacy and improved the survival of cancer patients. Studies of other immune checkpoint proteins, such as CTLA-4, TIM-3, LAG-3, and TIGIT, are likely to provide potential novel targets for immunotherapy. Here, we review the role of and recent advances in immune checkpoint blockade in common subtypes of PTCL, focusing on the anti-tumor immune responses to PD-1/PD-L1 blockers.

## 1 Introduction

Peripheral T-cell lymphomas (PTCLs) comprise a group of lymphoproliferative disorders that originate from mature T/NK cells and account for 25% of non-Hodgkin lymphomas (NHLs) in China and 10% of NHLs in Europe and the United States (Vose et al., 2008; Sun et al., 2012). There are 27 subtypes of PTCL according to the World Health Organization 2016 classification of lymphoid neoplasms (Swerdlow et al., 2016). Of these, extranodal NK/T-cell lymphoma, nasal-type (ENKTL), which is closely associated with Epstein-Barr virus (EBV) infection, is the most common in East Asian countries (Yoon et al., 2021). Other common subtypes of PTCL include PTCL, non-specific (PTCL-NOS), angioimmunoblastic T-cell lymphoma (AITL), and anaplastic lymphoma kinase (ALK)+/− anaplastic large cell lymphoma (ALCL) (Swerdlow et al., 2016; Liu et al., 2020). PTCLs are highly heterogeneous, aggressive, and, except for ALK+ ALCL and early-stage ENKTL, generally incurable. PTCLs are also associated with poor prognosis and are often resistant to cytotoxic chemotherapeutic drugs. The therapeutic options are limited, especially for relapsed or refractory (R/R) PTCL, for which new effective treatments are urgently needed.

Over the past few years, immune checkpoint inhibitors (ICIs) have become one of the most active areas in oncological research, and have fundamentally changed the paradigm for the clinical management of cancer patients (Pardoll, 2012). Programmed cell death protein 1 (PD-1), programmed cell death 1 ligand 1 (PD-L1), and cytotoxic T-lymphocyte-associated protein 4 (CTLA-4) inhibitors have been approved for the treatment of a variety of tumors, including lung cancer (Brahmer et al., 2015), melanoma (Tsai et al., 2014; Schadendorf et al., 2015), renal cancer (Motzer et al., 2015), and classic Hodgkin lymphoma (cHL) (Ansell et al., 2015). Several basic and clinical studies have suggested that PD-1/PD-L1-based ICIs have potential as important therapeutic tools for PTCL. Here, we systematically review current knowledge of the role and use of ICIs in PTCL.

## 2 Current Status of Peripheral T-Cell Lymphoma Treatment

Despite the different cellular origins and differences in their pathogenesis, the treatment of PTCLs has long followed the paradigm of the treatment for aggressive B-cell NHL owing to a lack of understanding of the pathogenesis of each subtype. Anthracycline-based regimens such as cyclophosphamide, doxorubicin, vincristine, prednisone (CHOP), or CHOP combined with etoposide (CHOEP), are the most used first-line treatments for PTCL other than NK/T-cell lymphoma. The CHOEP regimen can extend the overall survival (OS) of patients with PTCL to some extent, especially those aged ≤60 years and those presenting with ALK+ ALCL (Schmitz et al., 2010). In recent years, many studies have sought to improve the efficacy of CHOP-based chemotherapy, such as CHOP or CHOPE combined with gemcitabine, all of which have been unsuccessful (Liu et al., 2019; Kim et al., 2021). The 5-years OS associated with autologous stem cell transplantation (ASCT) consolidation therapy after chemotherapy with CHOP-based regimens is approximately 50%, while the 5-years progression-free survival (PFS) is only 45% (Abramson et al., 2014; Ellin et al., 2014). The recent identification and development of brentuximab vedotin (BV), an antibody–drug conjugate (ADC) consisting of a monoclonal antibody targeting CD30 coupled to the microtubule inhibitor monomethyl auristatin E (MMAE), represents a major milestone in the treatment of PTCL. The results of the ECHELON-2 study demonstrated that BV combined with CHP could provide clinically meaningful improvement as a first-line treatment for PTCL, achieving a 5-years PFS rate of 51.4% and a 5-years OS rate of 70.1% (Horwitz et al., 2022).

Treatment options for R/R PTCL are limited, and the median PFS and OS is usually less than 6 months (Mak et al., 2013). Allogeneic hematopoietic stem cell transplantation (HSCT) is a potential cure, but patients need to receive salvage chemotherapy and achieve complete response (CR) before transplantation (Mehta-Shah et al., 2020). Moreover, patients who relapse after transplantation or are not suitable for transplantation are often treated with single-agent palliative therapy. Four drugs are currently approved by the FDA for the treatment of R/R PTCL. BV monoclonal antibody, with an overall response rate (ORR) of 86% and duration of response (DOR) of 12.6 months in CD30-positive systemic R/R ALCL (Pro et al., 2017), may also benefit patients with CD30-positive PTCL-NOS (Horwitz et al., 2014). The other three drugs are the folic acid antagonist pralatrexate and two histone deacetylase (HDAC) inhibitors, romidepsin and belinostat. The efficiency of these single agents ranges from 25 to 30% and their DOR is limited (O'Connor et al., 2011; Coiffier et al., 2012; O'Connor et al., 2015). Work continues to identify new targets to improve treatment modalities.

Asparaginase-based chemotherapy regimens have significantly improved the survival of patients with ENKTL over the past decade. Treatment of localized ENKTL with radiotherapy or combination chemoradiotherapy is associated with a 5-years survival rate of approximately 70% (Kim et al., 2018), and patients with advanced and recurrent disease are usually treated with multi-drug combination chemotherapy, with a 5-years survival rate of approximately 40% (Kwong et al., 2012). Patients with R/R ENKTL who have failed asparaginase therapy do not have effective salvage therapy and have a poor prognosis, with a median OS of less than 6 months (Lin et al., 2020). Consequently, immunotherapies (ICIs, anti-CD30 and anti-CD38 monoclonal antibodies) against ENKTL- and EBV-related targets are increasingly being tried; however, no approved targeted or immunotherapeutic agent is currently available for the treatment of ENKTL.

## 3 Immunomodulatory Effects of CTLA-4 and PD-1

**FIGURE 1:**
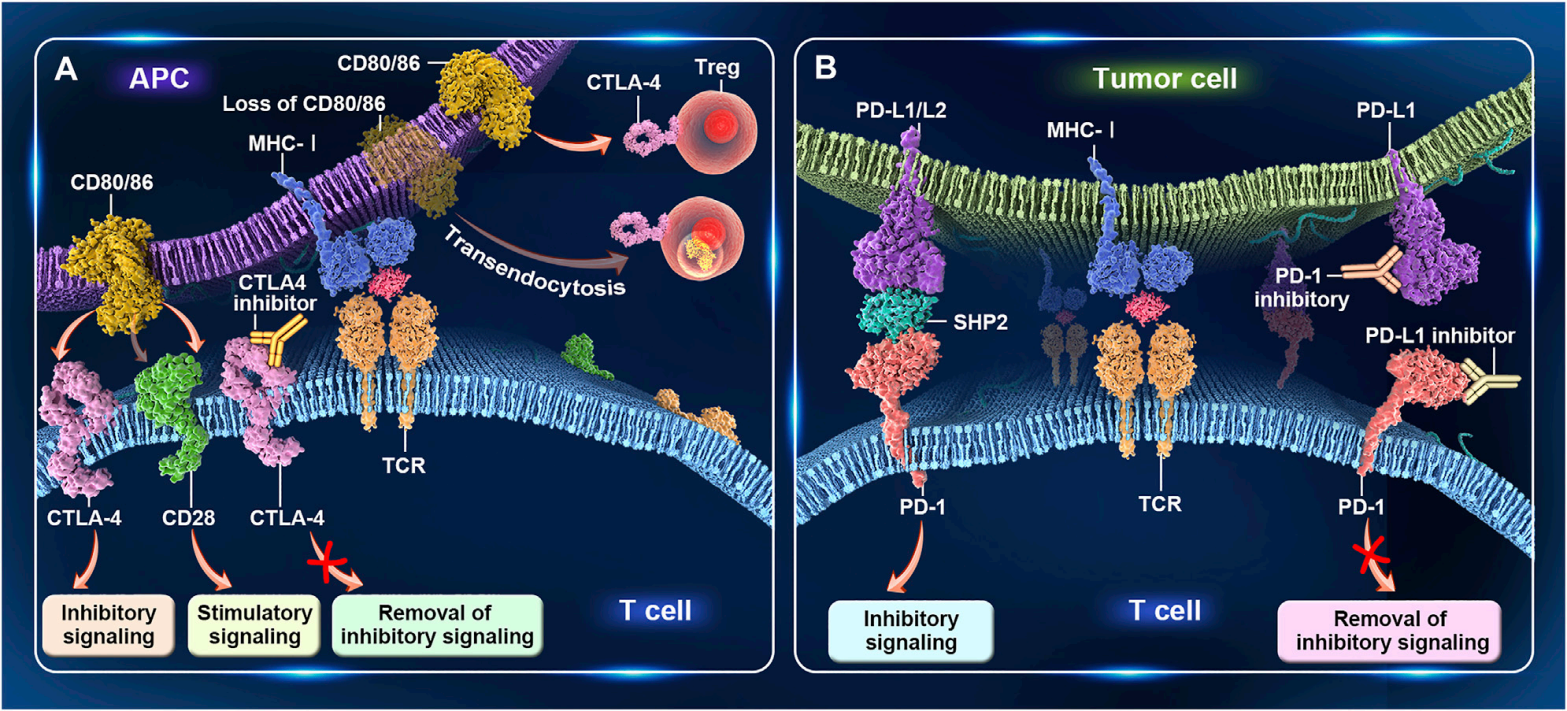
Immunomodulatory effects of CTLA-4 and PD-1. **(A)** CTLA-4 regulates T-cell activation. When CTLA-4 is overexpressed on activated T cells, it competes with CD28 for binding to CD80/86 on antigen-presenting cells (APCs), thereby inhibiting T-cell activation. Treg-specific deletion of CTLA-4 or the CTLA-4-mediated capture of CD80/86 from APCs through endocytosis leads to aberrant T-cell activity. **(B)** PD-1 regulates T-cell activation. PD-1 is mainly expressed on activated T cells. After binding to PD-L1/L2 on tumor cells, inhibitory signals transmitted to T cells. PD-1 antibodies block the interaction between PD-1 and PD-L1/PD-L2, resulting in the relief of these inhibitory signals, enhanced T-cell activity, and, finally, tumor cell lysis.

The T-cell receptor present on the surface of antigen-specific naive T cells recognizes the major histocompatibility complex on the surface of antigen-presenting cells (APCs), and this interaction represents the major signal of the immune response. CD28, expressed on the surface of naive CD4^+^ and CD8^+^ T cells, binds to the ligands CD80 (B7-1) and CD86 (B7-2) to generate a strong costimulatory signal, which is essential for the activation of antigen-specific naive T cells. Without this signal, T cells enter an unresponsive or hyporesponsive state, and lymphocytes cannot be functionally activated and do not survive for long periods (Greaves and Gribben, 2013). Immune system homeostasis is accomplished by the coordination of stimulatory and inhibitory signals. CTLA-4 is a homolog of CD28 expressed on activated T cells and regulatory T cells (Tregs), and has a higher binding affinity for CD80 and CD86 compared with CD28. When overexpressed, CTLA-4 competes with CD28 for binding to CD80 and CD86 on APCs, thereby inhibiting T-cell activation (Chen and Flies, 2013; Gardner et al., 2014). In addition, CTLA-4 regulates T-cell activation in an extracellular manner mediated by Tregs (Read et al., 2006; Friedline et al., 2009). CTLA-4 serves as a key functional molecule for Treg-mediated immune tolerance, and Treg-specific deletion of CTLA-4 leads to aberrant T-cell activation and autoimmunity (Jain et al., 2010). Additionally, CTLA-4 can rapidly capture CD80 and CD86 from APCs through endocytosis. Treg-derived CTLA-4 promotes Treg–APC binding and immune synapse formation, thereby providing a stable platform from which Tregs can deplete CD80/CD86 molecules on APCs through CTLA-4-dependent trogocytosis (Qureshi et al., 2011; Tekguc et al., 2021) (Figure 1A).

PD-1 (CD279) is another important immune checkpoint protein. This receptor is expressed mainly on activated T cells and, upon binding to its ligands PD-L1 (CD274) and PD-L2 (CD273) in tumor cells, transmits inhibitory signals to T cells, thereby helping to maintain a balance between immune tolerance and tissue damage (Dong et al., 1999). Moreover, an inadequate immune response leads to prolonged antigen stimulation, which results in PD-1 upregulation and T-cell exhaustion. Exhausted T cells initially acquire effector functions but subsequently become dysfunctional in response to chronic antigenic stimulation (Keir et al., 2008; Pauken and Wherry, 2015; Wherry and Kurachi, 2015). The overexpression of PD-L1 or PD-L2 in tumor cells or the tumor microenvironment can also lead to T-cell unresponsiveness or depletion and tumor cell evasion of immune surveillance. PD-1 antibodies inhibit the interaction between PD-1 and PD-L1/PD-L2, resulting in greater T-cell numbers, increased cytotoxicity, upregulation of cytokine production, and, ultimately, tumor cell lysis (Wang et al., 2018) (Figure 1B).

## 4 PD-1/PD-L Expression in Peripheral T-Cell Lymphoma

### 4.1 Expression of PD-1 and PD-L1/L2 in Tumor Cells

PD-1 and PD-L1 expression is commonly observed in PTCL cells and PD-1 is identified as a diagnostically relevant biomarker for AITL. The high expression of PD-1 in AITL was confirmed as early as 2007 (Shimauchi et al., 2007), and subsequent studies also found an increased number of PD-1-expressing cells in 93% of AITLs and 62% of follicles outside PTCL-NOS, leading to an immunosuppressive microenvironment (Krishnan et al., 2010). In a series of studies over recent years, Manso et al. (2021) evaluated PD-1 expression in 168 PTCL subtypes *via* immunohistochemistry using a cutoff value of 10% and reported that PD-1 was expressed in 61.6% (52/86) of AITL, 39.3% (22/56) of PTCL-NOS, and 13.3% (2/15) of ALK− ALCL cases. In contrast, PD-1 positivity was barely detectable in ALK+ ALCL. PD-1 expression was again confirmed to be higher in AITL than PTCL-NOS and ALCL. Kim S. et al. (2020) reported PD-1 and PD-L1 positive tumor cells in 76 PTCL cases (44 AITL and 32 PTCL-NOS) of 63.2 and 59.2%, respectively, with a cutoff value of 5%. In addition, Shi et al. (2021) assessed the efficacy and safety of the recombinant anti-PD-1 humanized monoclonal antibody geptanolimab (GB226) in patients with R/R PTCL while evaluating PD-L1 expression and its possible correlation with clinical outcomes. Of 81 patients with available PD-L1 expression data, 80 (98.8%) had PD-L1 expression of 1% or more, and 45 (55.6%) had PD-L1 expression of ≥50%. By subtype, the PD-L1 ≥50% rate was 78.9% (15/19) for ENKTL, 71.4% (5/7) for ALK+ ALCL, 38.5% (5/13) for ALK− ALCL, and 35.7% (10/28) for PTCL-NOS. Patients with PD-L1 expression of ≥50% derived more benefit from geptanolimab treatment. Panjwani et al. (2018) used immunohistochemistry to evaluate PD-L1 expression in tumor cells in 702 lymphoma biopsy specimens. The authors reported that 80% (12/15) of (ALK^+/−^) ALCL, 80% (16/20) of AITL, 39% (35/90) of ENKTL, and 26% (29/112) of PTCL-NOS tumor tissues stained positive for PD-L1. Studies have shown that PD-1 and PD-L1 are frequently expressed in PTCL and correlate with prognosis or staging (Sun et al., 2019; Qian et al., 2020).

Several of these studies reported that PD-1 expression was rarely or not detected (0–2.4%) in ENKTL cells (Kim et al., 2017; Zeng et al., 2019; Muhamad et al., 2020), while PD-L1+ tumor cells were observed in 56–79.9% of cases. However, results regarding the prognostic value of PD-L1 positivity have been inconsistent. PD-L1 positivity has been significantly associated with a low international prognostic index (IPI), normal serum lactate dehydrogenase, or a trend toward longer OS and PFS and improved overall survival (Kim et al., 2016; Jo et al., 2017). Alternatively, PD-L1 positivity was reported to be correlated with poor prognosis, lower event-free survival (EFS), significantly lower CR rates, or shorter PFS and OS (Bi et al., 2016; Zeng et al., 2019; Muhamad et al., 2020).

PD-L2 expression was reported to be significantly higher in B-cell tumors than in T/NK-cell tumors. One study identified PD-L2 expression in 78% (11/14) of primary mediastinal large B-cell lymphomas and 41% (20/49) of classic HL cases, but only 2.6% (4/152) of PTCL tumor cells expressed PD-L2, while all ENKTL tissues lacked PD-L2 expression (Panjwani et al., 2018). In contrast, another study showed significantly higher PD-L2 mRNA expression in the ENKTL cell lines SNK-6 and YTS compared with that in normal NK cells and a PD-L2 positivity rate of 63.3% (19/30) in 30 ENKTL tumor tissues (Han et al., 2014). A different study identified PD-L2 expression in 63.2% (31/49) of ENKTL cases with a cutoff value of ≥10%, while a subgroup analysis based on PD-L1/2 expression status showed superior EFS and OS in patients lacking PD-L expression on tumor cells. Despite the limited number of studies, it is evident that PD-L2 prevalence and distribution correlate with those of PD-L1; however, PD-L2 expression has also been detected in PD-L1-negative ENKTL (Muhamad et al., 2020). The inconsistent results reported for PD-L2 expression may be due to the different sensitivities of the anti-PD-L2 antibodies used or may be related to the geographical differences among ENKTL cases (United States of America, Asia, and Central America) in the above-mentioned studies.

In epithelial-derived malignancies, PD-L1 expression is associated with poor prognosis, regardless of the antibody used and the assessed cutoff values (Ghebeh et al., 2006; Thompson et al., 2006; Thompson et al., 2007), consistent with its role as a suppressor of tumor immunity. The prognostic impact of PD-L1 in lymphoma varies and its role as a prognostic indicator is complex. For consistency of results, studies investigating PD-1 and PD-L1/L2 expression and prognosis in lymphoma must consider several issues. First, there must be consistency in the treatment regimens of the included patients; notably, some studies on ENKTL have included patients whose regimens were not all based on asparaginase. Second, there is no standard scoring systems for interpreting PD-L expression in lymphoma, and the different primary antibodies used for immunohistochemical staining may affect study results. Currently, there are two FDA-approved complementary diagnostic kits and formal interpretation manuals for assessing PD-L expression (i.e., Ventana SP263 and SP142), both of which are currently being utilized primarily in non-small cell lung cancer (Liu et al., 2017; Scheerens et al., 2017); however, neither diagnostic test has been evaluated and systematically validated in lymphoma, although this could be attempted in the future. A recent study described a National Institute of Standards and Technology (NIST) standardized calibration tool with which IHC detection can be assessed using analytical parameters that include the limit of detection (LOD) and dynamic range of PD-L1. This tool helps to standardize the sensitivity of the assay and maintain the reproducibility of PD-L1 expression assessments between laboratories or on a weekly basis (Sompuram et al., 2022). Studies on ENKTL have often employed a 5% or 10% threshold to define positivity; however, the absence of a standard threshold could also explain the inconsistent findings (Diggs and Hsueh, 2017; Maleki Vareki et al., 2017). Finally, immune checkpoint pathways are complex, and the PD-1/PD-L1 pathway represents only one axis in an extremely complex regulatory network.

### 4.2 Soluble PD-L1

PD-L1 is expressed in two forms, namely, membrane-bound and soluble. Soluble PD-L1 (sPD-L1) has also been associated with immunosuppression and poor prognosis in malignancies such as multiple myeloma and DLBCL (Zhu and Lang, 2017). Currently, the enzyme-linked immunosorbent assay is the most widely used method for detecting the expression of sPD-L1 in the plasma or serum of patients. The results of many studies consistently show that sPD-L1 levels are higher in PTCL than in healthy volunteers and that plasma or serum sPD-L1 levels are positively correlated with PD-L1 expression in lymphoma tissue, suggesting that sPD-L1 levels are potential plasma biomarkers for predicting prognosis in patients with PTCL (Wang et al., 2016; Nagato et al., 2017; Shen et al., 2019; Zhang X. et al., 2019; Li et al., 2020). The same threshold normalization exists for sPD-L1 as for PD-L1.

### 4.3 PD-1+/PD-L1+ Tumor-Infiltrating Immune Cells

Tumor-infiltrating immune cells are an important component of the tumor microenvironment and have multiple functions. The best characterized of these cells are tumor-infiltrating lymphocytes (TILs) and tumor-associated macrophages. The presence of PD-1+ tumor-infiltrating immune cells is a prognostic indicator for many solid tumors, such as ovarian and rectal cancers (Zhang et al., 2003; Galon et al., 2006). Moreover, high expression of PD-1 and PD-L1 was found to be associated with a good prognosis, supporting that the expression of both molecules on tumor-infiltrating immune cells represents a strong immune response.

Many studies have shown a significant increase in the numbers of PD-1+ TILs in lymphomas, including cHL (Muenst et al., 2009), DLBCL (Ahearne et al., 2014; Kwon et al., 2016), and follicular lymphoma (Smeltzer et al., 2014). One study reported that PD-1 was expressed both in tumor cells and immune cells in 63.2% of 76 cases of PTCL (44 AITL and 32 PTCL-NOS) (Kim et al., 2020). Han et al. (2014) found that the number of PD-1+ TILs was significantly higher in nasal ENKTL tissue than in normal tissue and that PD-1 expression in TILs was downregulated after chemotherapy. Consistent with these findings, several other studies have confirmed that PD-1 is predominantly expressed in tumor-infiltrating immune cells in ENKTL, although the expression rate is relatively low, with PD-1+ TILs observed in only 11.4–20.5% of patients. Furthermore, no significant correlation was detected between PD-1+ TILs and prognosis (Jo et al., 2017; Muhamad et al., 2020), suggesting that ENKTL TILs express higher levels of PD-1 compared with tumor cells. Gene expression profiling of PD-1 positive and negative of mononuclear cells isolated from peripheral blood mononuclear cells derived from PTCL patients and healthy controls (obtained by incubation with PD-1 antibody and magnetic bead sorting) revealed that PD-1 positivity in patients with PTCL was associated with abnormal expression of genes related to the innate immune response, as well as with significantly upregulated expression of CTLA-4, reduced levels of IFN-γ secretion, and impaired cytotoxic activity (Zhang W. et al., 2019).

Relatively few studies have focused on the clinical role of PD-L1+ immune cells in the lymphoma microenvironment. One study found that 76.3% of PTCL tumor cells and immune cells were double-positive for PD-1 and PD-L1, and that PD-L1 expression was associated with poor prognosis in AITL patients (Kim et al., 2020). Another study reported that, in 79 cases of ENKTL, PD-L1 was expressed in 78.5% of TIL and was significantly associated with low IPI (Kim et al., 2016).

## 5 PD-1 Inhibitor Therapy

### 5.1 PD-1 Inhibitor Monotherapy

Nivolumab (trade name: Opdivo) is a fully humanized immunoglobulin (Ig) G4 anti-PD-1 monoclonal antibody. A phase I study evaluated its value in patients with various hematologic malignancies, including five with R/R PTCL. Partial response (PR) was achieved in two patients (Lesokhin et al., 2016). Another phase II study enrolled 12 patients with R/R PTCL—including six with AITL, three with PTCL-NOS, and one with ALK^−^ ALCL—half of whom had received ASCT. The ORR was 33%, including two CRs and two PRs, with median DOR, PFS, and OS of 3.6, 1.9, and 7.9 months, respectively. However, four patients experienced hyperprogression (defined as significant progression within one treatment cycle), and the study was terminated due to the high incidence of hyperprogression, moderate efficacy, and short DOR (Bennani et al., 2019). It has been suggested that this rapid disease progression may be related to PD-1 acting as a tumor suppressor in certain tumor types, further emphasizing the need for clinical studies to focus on biologically distinct subgroups (Wartewig et al., 2017; Ratner et al., 2018).

Pembrolizumab (trade name: Keytruda) was the second approved human IgG4 monoclonal antibody targeting PD-1. A phase II multicenter study that enrolled eight patients with R/R PTCL for treatment with this agent was stopped early after a pre-planned interim analysis for futility (Barta et al., 2019). Another study included seven patients with R/R ENKTL with a median number of lines of treatment of two or more before enrollment. All patients had used SMILE or SMILE-like regimens, and two had relapsed after receiving allogeneic HSCT. The ORR was 100% after a median of 7 weeks of treatment, with a median follow-up of 6 months. Five patients achieved sustained CR, including two that achieved CR in all parameters. Patients with high PD-L1 expression had a better prognosis. The only adverse event (AE) was the development of grade 2 cutaneous graft-versus-host disease in patients who received HSCT (Kwong et al., 2017). Another study also recruited seven patients with R/R ENKTL and achieved a median ORR of 57% after four courses, including two instances of CR and two of PR, with response duration, PFS, and OS of 4.1, 4.8, and 5.0 months, respectively. No direct relationship was observed between PD-L1 expression and treatment response. Treatment-related AEs occurred in 71.4% of patients, 28.6% of whom had grade 3 immune pneumonia which was managed safely. The remaining AEs were grade 1–2, and included anemia, neutropenia, and elevated aminotransferase levels (Li et al., 2018). Kim et al. (2019) evaluated the outcomes of 30 patients with R/R NHL treated with pembrolizumab, including 14 presenting with EBV-positive ENKTL. The ORR was 44%, five patients achieved CR, and one achieved PR. Patients with ENKTL with high PD-L1 expression in tumor cells (PD-L1-positive staining >50%) had a higher response rate than those with low PD-L1 expression (67% *vs*. 20%).

Sintilimab (trade name: Tyvyt) is a humanized IgG4 anti-PD-1 monoclonal antibody with a safety profile consistent with other approved PD-1 antibodies and was approved for R/R HL in China in 2018. A multicenter, single-arm, phase II study (ORIENT-4) was designed to investigate the efficacy and safety of sintilimab alone in Chinese patients with R/R ENKTL. A total of 28 patients were enrolled, 68% of whom were stage IV, while 89.3% had an ECOG score ≥1. Before enrollment, all the patients progressed after receiving an asparaginase-based regimen and a median of three lines of chemotherapy, 78.6% received radiation therapy, and 7.1% had relapsed after HSCT. With a median follow-up of 30.4 months, the ORR was 75% and the CR and PR rates were 21.4 and 53.6%, respectively. The 2-year OS rate was 78.6%, with a median OS not being reached. Most (71.4%) AEs were grade 1–2, with the most common being lymphopenia, which occurred in 42.9% of the patients, and no patient dropped out of treatment due to AEs (Tao et al., 2021).

Geptanolimab was evaluated in a phase II clinical trial (NCT03502629) to determine its efficacy and safety in patients with R/R PTCL and to assess whether there was a correlation between PD-L1 expression and clinical prognosis. The study was conducted in 41 centers in China and enrolled 102 patients, including 41 (40.2%) with PTCL-NOS, 23 (22.5%) with ENKTL, 12 (11.8%) with ALK− ALCL, 7 (6.9%) with ALK+ ALCL, and 19 (18.6%) with other PTCL subtypes. The 89 patients included in the analysis had a median follow-up of 4.06 months, the ORR was 40.4%, the CR rate was 14.6%, the PR rate was 25.8%, and the DOR was 11.4 months. Patients with PD-L1 expression ≥50% derived a greater benefit from the treatment (ORR, 53.3% *vs*. 25.0%; median PFS, 6.2 *vs*. 1.5 months). In total, 25.5% of patients experienced grade 3 and treatment-related AEs, most commonly lymphopenia and thrombocytopenia. Serious AEs were observed in 44.1% of patients, 18.6% of which were treatment-related. This study represented the largest prospective clinical trial evaluating the efficacy and safety of PD-1 inhibitors in patients with PTCL, with geptanolimab showing good promise and a manageable safety profile (Shi et al., 2021).

The main clinical results are shown in Table 1, according to it which can be seen that among PTCLs, PD-1 monotherapy shows the best promise in ENKTL, which may be related to its being a tumor species with high levels of PD-L1 expression. Studies investigating the mechanism underlying ENKTL pathogenesis reported that mutations in the *STAT3* gene lead to increased levels of STAT3 phosphorylation. Phosphorylated STAT3 binds the *PD-L1* gene promoter and positively regulates PD-L1 expression (Song et al., 2018). Furthermore, the EBV-encoded latent membrane protein 1 (LMP1) upregulates PD-L1 expression through the JAK/STAT and MAPK/NF-κB signaling pathways (Green et al., 2012; Bi et al., 2016). The results of the phase I trial to evaluate the efficacy and safety of geptanolimab (GB226) conducted in China in patients with R/R PTCL are very promising.

**TABLE 1:** Results of major clinical studies of PD-1/PD-L1 inhibitors for R/R PTCL.

Agent	Class	Single or Combination	Subtype	Trial Phase	*n*	Median Follow-Up	ORR (%)	CR	Median PFS	References
Nivolumab	PD-1 inhibitor	Single	R/R PTCL	I	5	44 weeks	40	0	14 weeks	Lesokhin et al. (2016)
Pembrolizumab	PD-1 inhibitor	Single	R/R ENKTL	—	7	6 months	100	71.4%	—	Kwong et al. (2017)
Pembrolizumab	PD-1 inhibitor	Single	R/R ENKTL	—	7	—	57	28.6%	4.8 months	Li et al. (2018)
Pembrolizumab	PD-1 inhibitor	Single	R/R ENKTL	—	14	—	44	35.7%	—	Kim et al. (2019)
Sintilimab	PD-1 inhibitor	Single	R/R ENKTL	II	28	30.4 months	75	21.4%	—	Tao et al. (2021)
Geptanolimab	PD-1 inhibitor	Single	R/R PTCL	II	102	4.06 months	40.4	14.6%	—	Shi et al. (2021)
Pembrolizumab	PD-1 inhibitor	Romidepsin	R/R PTCL	II	14	18 months	50	35.7%	—	Iyer et al. (2020)
Camrelizumab	PD-1 inhibitor	Apatinib	R/R PTCL	II	15	—	36.4	9.1%	5.47 months	Lin et al. (2020)
Toripalimab	PD-1 inhibitor	Chidamide, etoposide, and thalidomide	R/R ENKTL	II	3	—	100	66.7%	—	Du et al. (2020)
CS1001	PD-L1 inhibitor	Single	R/R ENKTL	II	29	5.55 months	44	36.0%	—	Huang et al. (2019)
Avelumab	PD-L1 inhibitor	Single	R/R ENKTL	II	21	15.7 months	38	24.0%	2.7 months	Kim S. J. et al. (2020)

R/R: relapsed or refractory; PTCL: peripheral T-cell lymphoma; ENKTL: extranodal NK/T-cell lymphoma, nasal-type; ORR: overall response rate; CR: complete response; PFS: progression-free survival.

### 5.2 PD-1 Inhibitor Combination Therapy

The strategy of combination therapy involving a PD-1 monoclonal antibody in combination with chemotherapy or targeted therapy also warrants evaluation. Although the pembrolizumab monotherapy trial was terminated after interim analysis, several trials are exploring its efficacy in combination therapy. A phase I/II study (NCT03278782) was conducted to evaluate the efficacy and safety of pembrolizumab in combination with the HDAC inhibitor romidepsin in the treatment of patients with R/R PTCL. Twenty evaluable patients had a phase I ORR of 44%, with three showing CR; and a phase II ORR of 50%, with five showing CR and two PR, with a median follow-up of 18 months. The most common grade ≥3 AEs were nausea, vomiting, and fatigue. Two patients experienced hyperprogression within the first 10 days of treatment, while four discontinued treatments due to immune-related AEs. These were grade 1 cytokine storm, grade 3 gastritis, grade 4 colitis, and grade 2 pneumonia, all of which were amenable to steroid treatment. PD-L1 expression was available for 13 of these patients, with a median PD-L1 expression of 10% in responders and 0 and 10%, respectively, in the two hyperprogressive patients. Patients who achieved CR had higher PD-L1 levels than those who achieved PR or stable disease (Iyer et al., 2019; Iyer et al., 2020). In addition, a phase I/II trial of pembrolizumab in combination with the HDAC inhibitor pralatrexate (NCT03598998) and a phase I trial of pembrolizumab in triple combination with pralatrexate and the epigenetic modifier decitabine (NCT03240211) in R/R PTCL are also underway.

Camrelizumab (trade name: AiRuiKa) is a human IgG4 monoclonal antibody targeting PD-1. Apatinib is a selective vascular endothelial growth factor receptor 2 tyrosine kinase inhibitor, and combining it with a PD-1 inhibitor has shown encouraging efficacy and manageable toxicity in patients with advanced hepatocellular carcinoma (Xu et al., 2019). A phase II study (NCT03701022) was designed to evaluate the efficacy of camrelizumab in combination with apatinib in R/R PTCL. As preliminary results, 14 patients with a median of three lines of treatment were enrolled, including four with PTCL-NOS, four with AITL, two with ALK− ALCL, and 4 with ENKTL. The ORR was 36.4%, the CR rate was 9.1%, and the median PFS was 5.47 months. Two patients with ALK− ALCL responded to treatment (one achieved CR and one PR). Most AEs were grade 1 to 2, with grade 3 AEs including thrombocytopenia (10.1%), neutropenia (4.5%), hypertension (4.5%), and rash (4.5%), and no AEs above grade 4 were observed. This multicenter study is ongoing (Lin et al., 2020).

Toripalimab (trade name: TuoYi) is a humanized monoclonal antibody targeting PD-1. Three patients with R/R ENKTL were recruited to receive toripalimab in combination with chidamide, etoposide, and thalidomide (PCET regimen). The ORR was 100%, and included two CRs and one PR. All three patients experienced grade 2–3 anemia and leukopenia (Du et al., 2020). The inclusion of chidamide in patients who progressed after pegaspargase-based chemotherapy or sintilimab monotherapy led to durable CR with mild toxicity, possibly because HDAC inhibitors exhibit pleiotropic immunomodulatory effects (Mazzone et al., 2017; Conte et al., 2018; Zhao and Zhang, 2019; Yan et al., 2020).

In addition, pembrolizumab combined with local radiotherapy in ENKTL is an option to be considered in older primary patients who are intolerant to chemotherapy (Asif et al., 2019), with a maximum duration of CR of more than 2 years reported in some cases (Klee et al., 2020). PD-1/PD-L1 inhibitors and radiotherapy have potential synergistic anti-tumor immunomodulatory effects, with radiotherapy providing a more suitable immune microenvironment for subsequent PD-1/PD-L1 inhibitor therapy, while PD-1/PD-L1 inhibitors can attenuate radioresistance and enhance distant effects (Chen et al., 2020).

## 6 PD-L1 Inhibitor Therapy

CS1001, a fully humanized IgG4 anti-PD-L1 monoclonal antibody, was evaluated for efficacy in patients with R/R ENKTL after the failure of prior asparaginase-based chemotherapy. Twenty-nine patients were enrolled, 75.9% of whom were stage IV. The median treatment duration was 11.7 weeks. With a median follow-up of 5.55 months, the ORR was 44%, the CR rate was 36%, the median DOR was not reached, and all patients who achieved CR remained in remission. Treatment-related AEs occurred in 72.4% of patients, with approximately 10.3% of AEs being ≥grade 3. Common AEs were fever (20.7%), elevated levels of thyroid-stimulating hormone (13.8%), leukopenia (13.8%), and rash (10.3%) (Huang et al., 2019). Preliminary data support the further exploration of PD-L1 inhibitors in R/R ENKTL, and combination strategies may help to further improve outcomes. Even in the asparaginase era, the poor prognosis of patients with advanced ENKTL remains poor. Accordingly, a prospective phase II study (NCT04127227) evaluating the combination of PD-L inhibitors with P-GEMOX for the treatment of primary advanced ENKTL is ongoing.

Avelumab (trade name: Bavencio) is another human IgG1 monoclonal antibody with enhanced antibody-dependent cell-mediated cytotoxic effects targeting PD-L1 (Boyerinas et al., 2015). A phase II study evaluated the efficacy of avelumab in patients with R/R ENKTL. Twenty-one patients were enrolled and the median follow-up was 15.7 months. The ORR was 38%, the CR rate was 24%, and the median PFS was 2.7 months. Treatment response was significantly correlated with PD-L1 expression in tumor tissue, and PD-L1 expression levels and immune subtype appear to help predict the response to avelumab (Kim S. J. et al., 2020). There are also several ongoing clinical trials evaluating the efficacy of avelumab in PTCL (NCT03046953, NCT03905135) and ALCL (NCT03905135).

Durvalumab (trade name: Imfinzi) is a human IgG1 anti-PD-L1 monoclonal antibody, and clinical trials are currently underway investigating the use of durvalumab alone or in combination with lenalidomide for the treatment of R/R PTCL (NCT03011814), in combination with pramipexole, romidepsin, and/or azacitidine (NCT03161223) for the treatment of T-cell lymphoma.

Clinical trial data for PD-L1 inhibitors in the treatment of PTCL subtypes are scarce and, except for ENKTL, are as yet unpublished. Despite this, their potential efficacy is supported by basic trials. Alisertib is a selective inhibitor of aurora kinase (AK), a key regulator of mitosis and an inducer of PTCL proliferation. Alisertib displays highly synergistic effects with PI3Kα inhibitors or vincristine *in vitro*. Alisertib treatment significantly reduced PD-L1 and NF-κB expression, inhibited the phosphorylation of AKT, ERK1/2, and AK, and increased the rate of apoptosis. In a PTCL mouse xenograft model, tumor growth inhibition following alisertib treatment was only approximately 30%, while anti-PD-L1 monotherapy was ineffective; however, alisertib combined with anti-PD-L1 treatment resulted in >90% growth inhibition, indicative of a synthetic lethal interaction. In addition, the combination of a pan-PI3K inhibitor (PF-04691502), alisertib, vincristine, and anti-PD-L1 treatment elicited a growth inhibition of 100%. Overall, the treatment was well tolerated in mice, and co-targeting PD-L1, AK, and PI3K may represent a reasonable and novel strategy for the treatment of PTCL (Islam et al., 2017).

## 7 Other Immune Checkpoint Inhibitors

Ipilimumab (trade name: Yervoy), a CTLA-4 inhibitor, was first introduced for the treatment of metastatic melanoma while tremelimumab, another CTLA-4 inhibitor, was initially explored for its efficacy in malignant mesothelioma. Overall, the effect of CTLA-4 inhibitors in PTCL is not well characterized. One study identified CTLA-4/CD28 fusion genes in 58% of AITLs, 23% of PTCL-NOS, and 29% of ENKTLs by reverse transcription-polymerase chain reaction and Sanger sequencing (Yoo et al., 2016). The fusion gene encodes the extracellular structural domain of CTLA-4 and the cytoplasmic domain of CD28, a combination that can convert inhibitory signals for T-cell activation into stimulatory ones. Ectopic expression of the fusion transcript in Jurkat and H9 T lymphocytes led to accelerated cell proliferation and the phosphorylation of AKT/ERK, thereby activating downstream oncogenic pathways. Further deep sequencing revealed a 30% frequency of CTLA-4/CD28 fusions in PTCL samples, suggesting that CTLA-4/CD28 gene fusions may be important in the pathogenesis of PTCL and represent a potential target for anti-CTLA-4 immunotherapy. In another study (Ma et al., 2016), the growth of a humanized lymphoma in mice induced by the injection of EBV (strain 81)-infected human cord blood was assessed following the simultaneous blockade of PD-1 and CTLA-4. This strategy led to a significant reduction in lymphoma growth, an increase in the number of tumor-infiltrating CD4^+^ and CD8^+^ T cells, and the promotion of EBV-specific T-cell responses. This study showed that PD-1/CTLA-4 blockade can enhance the ability of cord blood T cells to control EBV-induced lymphoma growth and suggested that PD-1/CTLA-4 blockade may be useful in the treatment of some human EBV-induced diseases.

T-cell immunoglobulin and mucin domain-containing protein-3 (TIM-3), lymphocyte activation gene-3 (LAG-3), and T-cell immunoglobulin and ITIM domain (TIGIT) were recently identified as immune checkpoint proteins that regulate immune function and have been associated with tumor development. TIM-3 is a negative regulator of T-cell function expressed on the surface of CD4^+^ helper T cells and CD8^+^ cytotoxic T cells and has been shown to promote T-cell depletion during tumor progression and chronic viral infections. LAG-3 is a negative immunomodulator that is mainly distributed in activated T cells and NK cells (Huard et al., 1997). TIM-3 and LAG-3 were found to be expressed in ENKTL with a 95% positivity rate (39/41), while high TIM-3 expression was reported to be an independent poor prognostic factor for PFS (Feng et al., 2018). Moreover, the TIM-3 positivity rate was reported to be 8% in PTCL-NOS (Murga-Zamalloa et al., 2020). These observations suggest that TIM-3 and LAG-3 may be potential targets for lymphoma therapy. TIGIT is highly expressed in Tregs, follicular helper T cells, effector T cells, and NK cells and acts as a co-immunosuppressor (Yu et al., 2009; Manieri et al., 2017). Indeed, the immune checkpoint receptors TIM-3, LAG-3, TIGIT, and PD-1 on CD4^+^ and CD8^+^ T cells have co-expression and co-regulatory properties (Chihara et al., 2018; DeLong et al., 2019). The combined blockade of the TIM-3 and PD-1 pathways has shown significant efficacy in solid (Sakuishi et al., 2010; Ngiow et al., 2011) and hematological (Zhou et al., 2011) tumors in basic studies. Accordingly, clinical trials on combined TIM-3 and PD-1 blockade are currently underway, mainly for solid tumors. Results are expected in the near future.

## 8 Biomarkers for Predicting the Efficacy of Immune Checkpoint Inhibitors

In addition to PD-1/PD-L1 expression, the tumor mutational burden (TMB) is also a relevant predictor of the response to immune checkpoint blockade (Klempner et al., 2019). The mutational profile of human malignancies has been extensively analyzed (Alexandrov et al., 2013), and tumors with increased mutational load, such as melanoma and non-small cell lung cancer, have prominent responsiveness to ICIs. Conversely, tumors with a low mutational load, such as acute lymphoblastic leukemia and acute myeloid leukemia, tend to be resistant to immune checkpoint blockade (Klempner et al., 2019). The response of patients with a high TMB to ICIs is thought to involve the stimulation of antitumor immune responses with neoantigens expressed in tumors rather than in normal cells. In PTCL, the mutational load varies significantly by subtype (Heavican et al., 2019), and in some cases approaches that of melanoma (Lawrence et al., 2014). PTCL-NOS patients with *p53* mutation have a greater TMB compared with PTCL-NOS patients without *p53* mutation. In conclusion, although not systematically reported so far, the mutational load of PTCL may vary by subtype, histology, and stage.

A recent study identified structural rearrangements in the *PD-L1* gene as potential predictive biomarkers of ENKTL for pembrolizumab responders by whole-genome sequencing (Lim et al., 2020). The authors used three immunohistochemical markers—Foxp3, PD-L1, and CD68—to classify ENKTL into the following four immune microenvironment subgroups: An immune-tolerant group, characterized by elevated numbers of Tregs; two immune escape (A and B) groups, characterized by high cytotoxic T-cell counts, high PD-L1 expression levels, and low Treg counts; and an immune-silenced group, where immune responses were depleted and most patients were in advanced stages, and which was associated with the poorest prognosis among the subgroups (Cho et al., 2020). However, large sample studies are needed to further support these results.

## 9 Conclusion and Future Prospects

PTCLs are highly heterogeneous and relapsed and refractory cases are common among affected patients. The rapid development of basic and clinical research has led to the emergence of immunotherapy, which has provided additional benefits to patients. The expression of PD-1 and PD-L1 can be detected in both PTCL cells and immune cells in the tumor microenvironment, and is the basis for the preclinical and clinical exploration of antibodies targeting these proteins. However, there are differences in PD-1/PD-L1expression among subtypes, and inconsistencies of clinical significance related to prognosis. These findings reflect the unique biology of PTCL, which should be considered when designing subsequent studies. The mechanisms involved should also be explored in depth. The use of PD-1/PD-L1 inhibitors in PTCL is encouraging based on current knowledge, especially for ENKTL, with patients with high PD-L1 expression benefiting the most. Further studies should focus on the development of biomarkers to better predict responders and allow more precise and individualized treatment. In addition, AEs for PD-1/PD-L1 inhibitors should not be ignored; nonetheless, in general, both drug-related toxicity and immune-related AEs are moderately well tolerated. For other immune checkpoint proteins, such as CTLA-4, TIGIT, TIM-3, and LAG-3, their evaluation in PTCL is still at the preclinical stage and needs to be validated *via* relevant clinical trials. Combinations of different ICIs and the combination of ICIs with cell therapy (e.g., PD-1/PD-L1 inhibitors + CAR-T cell therapy) may further enhance anti-lymphoma activity in the future. We believe that more studies will provide the best strategy for patients and improve their prognosis.
